# Accuracy of relocation, evaluation of geometric uncertainties and clinical target volume (CTV) to planning target volume (PTV) margin in fractionated stereotactic radiotherapy for intracranial tumors using relocatable Gill‐Thomas‐Cosman (GTC) frame

**DOI:** 10.1120/jacmp.v12i2.3260

**Published:** 2010-12-28

**Authors:** Saikat Das, Rajesh Isiah, B. Rajesh, B. Paul Ravindran, Rabi Raja Singh, Selvamani Backianathan, J. Subhashini

**Affiliations:** ^1^ Department of Radiation Oncology Christian Medical College Vellore India

**Keywords:** Gill‐Thomas‐Cosman frame, stereotactic radiotherapy, depth helmet, systematic error, random error, quality assurance

## Abstract

The present study is aimed at determination of accuracy of relocation of Gill‐Thomas‐Cosman frame during fractionated stereotactic radiotherapy. The study aims to quantitatively determine the magnitudes of error in anteroposterior, mediolateral and craniocaudal directions, and determine the margin between clinical target volume to planning target volume based on systematic and random errors. Daily relocation error was measured using depth helmet and measuring probe. Based on the measurements, translational displacements in anteroposterior (z), mediolateral (x), and craniocaudal (y) directions were calculated. Based on the displacements in x, y and z directions, systematic and random error were calculated and three‐dimensional radial displacement vector was determined. Systematic and random errors were used to derive CTV to PTV margin. The errors were within ± 2 mm in 99.2% cases in anteroposterior direction (AP), in 99.6% cases in mediolateral direction (ML), and in 97.6% cases in craniocaudal direction (CC). In AP, ML and CC directions, systematic errors were 0.56, 0.38, 0.42 mm and random errors were 1.86, 1.36 and 0.73 mm, respectively. Mean radial displacement was 1.03 mm ± 0.34. CTV to PTV margins calculated by ICRU formula were 1.86, 1.45 and 0.93 mm; by Stroom's formula they were 2.42, 1.74 and 1.35 mm; by van Herk's formula they were 2.7, 1.93 and 1.56 mm (AP, ML and CC directions). Depth helmet with measuring probe provides a clinically viable way for assessing the relocation accuracy of GTC frame. The errors were within ± 2 mm in all directions. Systematic and random errors were more along the anteroposterior axes. According to the ICRU formula, a margin of 2 mm around the tumor seems to be adequate.

PACS number: 87.55.‐x

## I. INTRODUCTION

Geometric uncertainties are introduced during the process of treatment of solid tumors by external beam radiotherapy. Stereotactic radiotherapy is a highly precise form of radiotherapy which is sensitive to setup error, as even a small error can have a huge impact on the desired dose distribution of the target tissue or the organ at risk. This may result in lower dose to the target or higher than the desired dose to the organs at risk. Errors in fractionated stereotactic radiotherapy are caused by relocation uncertainties of the stereotactic frame before each treatment sessions including intra‐ and interobserver variation of readings, and linear accelerator, laser and treatment setup related inaccuracies.

Various immobilization devices like Gill–Thomas–Cosman relocatable stereotactic head frame (GTC frame), BrainLAB device and thermoplastic ray casts have been used for fractionated stereotactic radiotherapy. These are highly reproducible and allow keeping a narrow margin between the CTV and the PTV, minimizing the volume of normal tissue irradiated around the target.^(1)^ In addition to these devices, various other immobilization methods have been reported in literature. For example, the Heidelberg group developed a relocatable stereotactic radiotherapy system using a mask substantially more rigid than a conventional thermoplastic mask, together with a stereotactic frame and localizer.^(2)^ Using this immobilization device along with a photogrammetric apparatus to detect displacements, they reported standard deviations of reproducibility in anteroposterior (0.9 mm), mediolateral (0.6 mm) and craniocaudal (1.3 mm) directions. Hamilton et al.^(3)^ assessed the reproducibility of a head shell and frame system, akin to the Heidelberg type, using radio‐opaque spheres embedded in a custom mouthpiece which was independent of the mask and frame. The reproducibility was analyzed using portal films on which images of the spheres could be seen. In 104 setups on 12 patients they found a mean deviation in the position of the isocenter of 1.8 mm, with a standard deviation of 1.4 mm. The maximum displacement seen was 6 mm. In an interim analysis published by Bednartz et al.^(4)^ comparing GTC frame with BrainLAB ray cast (n=37), the mean displacement for GTC frame is 1.93±0.98 mm and for the BrainLAB system it is 3.19±2.06 mm. A study by Jaywant^(5)^ showed the accuracy of the GTC frame to be 1 mm, and that it is also affected by dentures and dental disposition. In another study by Burton et al.^(1)^ in Addenbrooke's hospital, there was a skewed distribution for displacements in x, y, z axes for GTC frame and the mean displacement vector was 1.2 mm. In 92% of the patients in this study the displacement vector was less than 2 mm and only in 3% of patients was it more than 2.5 mm.

We describe a clinically viable method of checking errors in a couch‐mounted stereotactic frame in a busy radiotherapy department where fractionated stereotactic radiotherapy is incorporated as a routine treatment procedure. First the accuracy and geometric uncertainties of relocation of GTC frame were determined. The data were used to determine the margin for CTV to create PTV for treatment planning. We treat intracranial lesions of sizes up to 4 cm and these commonly include pituitary adenoma, craniopharyngioma, meningioma, optic glioma and schwannoma.

## II. MATERIALS AND METHODS

A total of 10 patients (age range 6 to 50 yrs, male/female ratio 1:1) with intracranial tumors planned for fractionated stereotactic radiotherapy using GTC frame were included in this prospective trial. These intracranial lesions included craniopharyngioma (4), pituitary adenoma (4), meningioma (1) and optic glioma (1). The dose delivered was in the range of 54 to 56 Gy with 1.8 to 2 Gy per fraction.

### A. GTC frame and measurement of relocation using depth helmet

**Figure 1:**
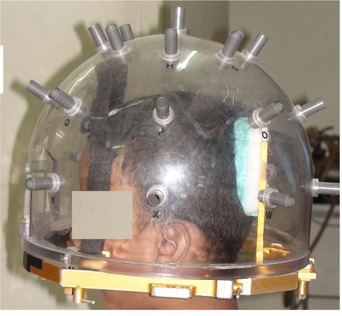
GTC frame with depth helmet fitted on a patient with dental occlusion and occipital pad. Radial measurements are taken with the help of the measuring probe at the depth helmet portal.

The basic design of the GTC frame was developed by Gill and his colleagues in London (Gill SS 1987, UK patent application N 8728150). The depth helmet was an additional device designed and added to the frame in Boston, USA and it was called the depth confirmation helmet. The GTC frame consists of a base ring (made of aluminum alloy), oral appliance, occipital pad and Velcro straps. The oral appliance is formed by taking a dental impression of the teeth. The occipital pad is an impression of the posterior skull encompassing the occipital protuberance. The oral appliance and the occipital pad are customized for each patient, and are both rigidly fixed to the frame. Together with the oral appliance and the occipital pad, the Velcro straps are used to maintain the position of the frame on the patient. The lower limit of the frame is at the base of the skull just below the soft palate. The individualized components (oral appliance and occipital pad) were custom made in the mold room using Aquasil soft putty (manufactured by Dentsply, Germany). The depth helmet attaches securely to the head frame fitted to the patient's head. The helmet is a Perspex hemisphere with a series of 24 evenly‐spaced measuring portals. Using a metal measuring probe inserted through the portal, a set of measurements was obtained from the outer edge of the helmet to the scalp (Fig. 1). Measurements were taken at the first visit when mouth bite and occipital pad were fixed and on two subsequent days for verification prior to doing the planning CT scan. The measurements taken at the time of the planning CT scan were used as baseline.^(6)^ Relocation accuracy was checked by depth helmet measurement before each treatment episode. The radial measurements obtained from depth helmet measurements can be converted to translational measurement in mediolateral (x), craniocaudal (y) and anteroposterior (z) axes and a three‐dimensional displacement vector can be calculated. The frame is considered to have acceptable reproducibility provided that the difference from the baseline measurement set are no worse than one reading > 2 mm, or three readings > 1.5 mm. When these tolerances are not met, the frame needs to be redone and measurements repeated.^(1)^

### B. Study of the accuracy and precision of stereotactic frame using head phantom

**Figure 2:**
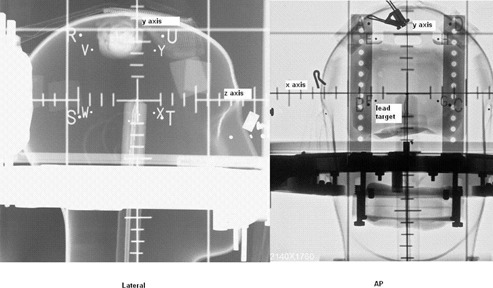
Computed radiographic film of the head phantom fitted with GTC frame and the angiographic localizer box. Small lead target is placed at the center Lateral (left) and AP (right) images are taken and mismatch of the target compared to the fiducials are found. The direction of x, y and z axes are also illustrated.

Phantom study was done to check the accuracy of relocation of the stereotactic frame using a head phantom made of Perspex. The set up is shown in Fig. 2. A custom‐made dental impression akin to that used clinically was fitted to Perspex bite block which was attached to the lips of the head phantom simulating the clinical situation. Similarly an occipital pad was made and fitted over the occipital area. The stereotactic frame was strapped firmly to the head phantom using Velcro straps.

The depth helmet was fitted with the frame and measurements were obtained with the measuring probe, as done clinically. To determine the precision of measurement and interobserver variations, three different radiation technologists who had been trained in SRT treatment measured the depth helmet readings using the head phantom. One technologist repeated the entire procedure on three separate occasions to give an idea of intraobserver variation.

In order to study the accuracy of relocation and resolution of the stereotactic frame, a small lead target was kept within the central chamber of the head phantom. An angiographic localizer box (with four fiducials on each side) was fitted to the frame and orthogonal images were taken (AP and Lateral). The experiment was repeated three times. The fiducials and the target were digitized, and target coordinates were derived from the above fiducial points using software developed in‐house, and the mismatch was calculated.

### C. Errors related to the treatment machine geometry

Mechanical errors can also arise due to the uncertainties in geometry of the linear accelerator, laser alignment and treatment setup related inaccuracies. For these, various quality checks are in place.

### D. Laser alignment with stereotactic frame

For characterization of error due to the laser alignment with couch‐mounted stereotactic frame isocenter, a QA tool consisting of crosshair which is fitted to the center of the angiographic localizer box was used. The angiographic localizer box was fitted to the couch‐mounted frame. Isocenter crosses marked on the setup plates and the crosshair were aligned with the lateral and ceiling lasers, and displacement measured at various gantry positions (0°, 90° and 270°) and couch positions (90°, 270°). The average of daily recordings of displacement for a month was taken, resulting in a total of 22 readings.

### E. Laser alignment with radiation isocenter

Isocentric shift is checked weekly using the Winston‐Lutz Alignment QA test tool consisting of a lead ball of 6 mm in diameter fixed to the center of the angiographic localizer box and positioned at the isocenter of the linear accelerator. Field size used was $1.2\times 1.2\;{\text{cm}}^{2}$ at various gantry positions (0°, 90° and 270°) and couch positions (90°, 270°). Electronic portal images were acquired, and isocentric displacements were digitally analyzed.

### F. Calculation of systematic and random error

The radial measurements obtained from the depth helmet measurements were used to derive the translational errors in the mediolateral (x), craniocaudal (y), anteroposterior (z) axes. For this purpose, a spreadsheet was used (obtained from K. E. Burton Department of Oncology, Addenbrooke's Hospital, Cambridge, with kind permission) to calculate the translational displacements in mediolateral, craniocaudal and anteroposterior directions. (For detailed method, please refer to Burton et al.^(1)^). A shift towards right direction of the patient in mediolateral axis, cranial direction in craniocaudal axis and anteriorly in anteroposterior axis was taken as positive. Displacements obtained on each day were compiled and arithmetic mean was calculated. This gives the systematic error for each patient. Systematic error was subtracted from total error to obtain random error for each treatment episode. The standard deviation of individual systematic error gives the population systematic error (Σ) and standard deviation of individual random errors gives the population random error (σ).

### G. Calculation of CTV to PTV margin

Systematic and random errors were used to calculate CTV to PTV margin.^(7)^ Several methods have been proposed in the literature to calculate CTV to PTV margin. We calculated CTV to PTV margin using ICRU 62(Σ+0.7σ) ^(8)^ Stroom's (2Σ+0.7σ) ^(9)^ and Van Herk's (2.5Σ+0.7σ) ^(^ ^10^ ^,^ ^11^ ^)^ formula. ICRU‐50 does not recommend adding all uncertainties linearly because the margin would become too large.^(7)^ As an alternative, the root sum of squares of setup error (ext) and organ motion (int) is suggested (e.g., $\sigma_{\text{total}}=\surd {\sigma_{\text{ext}}}^{2}{+\sigma_{\text{int}}}^{2}$). ICRU 62 mentions that systematic and random uncertainties should ideally be added in quadrature to obtain one standard deviation (i.e., ${\text{SD}}_{\text{total}}=\surd \Sigma_{2}+\sigma_{2}$) which should then be used to obtain a margin.^(8)^ However, this approach assumes that systematic and random errors have equal effects on patient's dose distribution. Studies have shown that the effect of systematic and random errors is different from a dosimetric point of view.^(9)^ All fractions are influenced by the systematic errors in an identical manner, whereas random error will be different for different fractions. Several authors have used the differential effects of systematic and random error in margin calculations.^(^ ^9^ ^,^ ^10^ ^,^ ^11^ ^)^ When allowing a fixed reduction of the minimum cumulative dose (i.e., to 95%), the margins of the random errors is small (i.e., 0.7 σ).^(12)^

### H. Calculation of radial error

The length of three‐dimensional radial displacement vector was calculated from the measurements in x, y and z directions, by square root of the summation of squares. The spreadsheets were compiled for each patient and pooled data were obtained for the population.

## III. RESULTS

### A. Accuracy of relocation of GTC frame

The accuracy of relocation of the stereotactic frame was studied using the orthogonal images (AP and Lateral) of the head phantom with a lead target inside. Using the fiducials of the angiographic localizer box, the error of target localization was 0.41 mm ± 0.16 mm. In the phantom experiment, the values of the standard deviation, which is an estimate of error of target coordinate localization in anteroposterior, mediolateral and craniocaudal axes, were 0.11, 0.21 and 2.68, respectively.

The precision of relocation of the GTC frame is also affected by interobserver and intraobserver variation. The interobserver variations were 0.05 mm ± 0.14 mm in mediolateral axis, 0.76 mm ± 0.17 mm in anteroposterior axis, and 0.07 mm ± 0.07 mm in craniocaudal axis. The intraobserver variations were 0.31 mm ± 0.11 mm, 0.32 mm ± 0.13 mm, and 0.51 mm ± 0.17 mm, respectively.

The error of laser alignment with the frame isocenter for various gantry and couch angles ranged from 0.2 mm ± 0.3 mm to 0.6 mm ± 0.2 mm. The mean error was 0.32 mm ± 0.18 mm. This is an average of a month's worth of daily QA reading (22 readings). The error of isocentric shift measured using Winston Lutz QA tool ranged from 0 to 1 mm (maximum), with mean value of 0.65 mm ± 0.32 mm (an average of 25 readings in different couch and gantry position).

### B. Evaluation of geometric uncertainties for GTC frame

**Figure 3:**
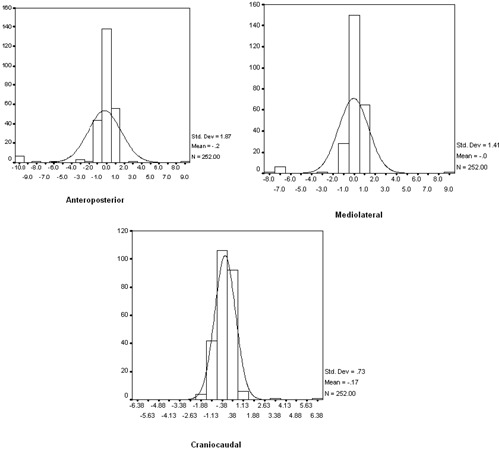
Distribution of random errors in anteroposterior, mediolateral and craniocaudal directions is normally distributed. As is seen from the figure, random error is more in anteroposterior axis.

Table 1 shows mean displacements (individual systematic error) calculated from the depth helmet measurements in mediolateral, anteroposterior and craniocaudal directions, and the mean vector displacements for each patient. Mean displacement ranged from 0.02 to 0.99 mm. The mean error in AP direction was 0.38 mm, ML direction 0.15 mm and CC direction was 0.17 mm. The errors were within ± 2 mm in mediolateral direction in 99.6% of cases, in 99.2% of cases in anteroposterior direction and in 97.6% of cases in craniocaudal direction. It was highest in the anteroposterior direction (0.38 mm) along yz plane. The population systematic error and random error were also more in this axis: 0.56 mm and 1.86 mm, respectively. The random errors calculated in each direction for the entire population was normally distributed (Fig. 3) in all three axes. This graph also illustrates that though random errors are normally distributed, they tend to be more along the yz plane.

**Table 1:** Systematic, random errors and CTV to PTV margin in different directions.

		*Error (mm)*			
*Patient No.*	*No of readings/day*	*Ant‐Post (z)*	*Mediolateral (x)*	*Craniocaudal (y)*	*Vector Length (r)*
1	24	0.45	0.02	−0.49	0.78
2	29	−0.99	0.20	0.40	1.72
3	26	0.99	0.46	−0.40	1.24
4	26	0.29	0.71	0.21	1.06
5	26	−0.76	−0.76	−0.23	1.20
6	24	0.06	0.36	−0.36	0.61
7	20	0.16	0.12	0.21	0.62
8	25	0.17	0.17	−0.42	0.80
9	27	−0.07	0.06	0.26	1.15
10	25	0.08	0.23	−0.91	1.19
Population mean error		0.38	0.15	−0.17	1.03SD=0.34
Population systematic error (Σ)		0.56	0.38	0.42	
Population random error (σ)		1.86	1.36	0.73	
CTV to PTV margin (ICRU)		1.86	1.45	0.93	
CTV to PTV margin (Stroom)		2.42	1.74	1.35	
CTV to PTV margin (Van Herk)		2.7	1.93	1.56	
Errors between +2mm and −2 mm		99.2%	99.6%	97.6%	
Errors more than +2 mm or less than −2 mm		0.8%	0.4%	2.4%	

Σ = population systematic error, σ = random error, SD = standard deviation

### C. Determination of CTV to PTV margin

The margin calculated by ICRU formula was 1.45 mm in mediolateral axis, 1.86 mm in anteroposterior axis and 0.93 mm in craniocaudal axis. The margin calculated by Stroom's formula^(9)^ was 2.42 mm in anteroposterior axis, 1.74 mm in mediolateral axis, and 1.35 mm in craniocaudal axis. The margin calculated by van Herk's formula ^(12)^ was 2.7 mm in anteroposterior axis, 1.93 mm in mediolateral axis, and 1.56 mm in craniocaudal axis. From the results it is evident that CTV to PTV margin calculated is more in anteroposterior direction (i.e, yz plane).

**Figure 4:**
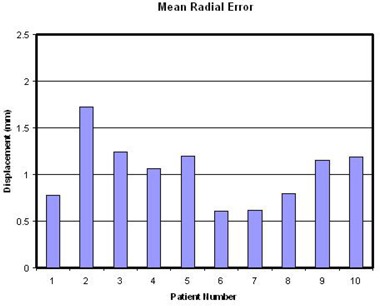
Mean radial displacements calculated for 10 patients from the displacements in AP, ML and CC axes. The values are well encompassed in a 2 mm margin and mean value is 1.03 mm ± 0.34 mm.

The mean vector displacement or radial error for the population is 1.03 mm (± 0.34 mm). This would represent a combined margin based on the errors calculated in the three axes. Figure 4 shows the mean vector displacement for all ten patients graphically. This shows that radial displacement is less than 2 mm.

## IV. DISCUSSION

Stereotactic radiotherapy requires a high degree of precision to deliver adequate dose to the target site, which requires accurate positioning of the stereotactic head frame. The margin should be optimum to avoid geographic miss or excess dose to the normal structures. Phantom based experiments show that the experimental error of accuracy of relocation is around 0.5 mm. Intra‐ and Interobserver variations contributes about 0.5 mm to 0.7 mm. Geometric uncertainty of the linear accelerator, stereotactic frame and lasers contribute another 0.3 to 0.6 mm. The error derived from depth helmet measurements comprises placement error of the frame, intra‐and interobserver variations, and relocation error when the frame is fitted to patient's head. Depth helmet measurements prior to treatment sessions show that in 97%–99% of cases, the error is within 2 mm.

Systematic and random errors have different effects on dose distribution. Systematic errors are persistent and constant throughout the treatment, whereas random uncertainties assume a different magnitude and direction for every treatment fraction. Where systematic error results in shift of the dose distribution, random errors lead to blurring. In the case of systematic errors, all fractions are equally affected leading to a very serious problem due to the shifting of the dose distribution, as the CTV may shift out of the high‐dose region. However, random error may take place everyday and small dose variation will lead to blurring causing decrease of the dose at the high‐dose region near the edge.^(13)^

In a study by Burton et al.^(1)^ errors in AP, ML and CC directions were reported to be 0.1 mm, 0.1 mm and 0.4 mm, respectively. Bednarz et al.^(4)^ reported errors of the magnitude of 0.34 mm in mediolateral direction, 0.55 mm in craniocaudal direction and 0.45 mm in AP direction, based on their study using kilovoltage images for calculation of displacements. Using relocatable TALON frame Salter et al.^(14)^ reported displacements ranging from 0.51 to 0.95 mm and they found the error to increase during the course of radiotherapy. The authors used repeated CT imaging to compare positional accuracy. Using Laitinen stereotactic localizer and head holder, Kalapurakal et al.^(6)^ reported errors ranging from 0.8 to 1.7 mm. In studies by Warrington et al.^(15)^ at Royal Marsden hospital using depth helmet measurement, the authors reported the inaccuracy to be around 3 mm and may go as high as 4 mm.

CTV to PTV margin calculated by ICRU formula was with in 2 mm in all directions. A CTV to PTV margin of 2 mm (as derived by Stroom's and van Herk formula) is adequate to account for the setup errors in all directions, except in the anteroposterior direction (yz plane). The radial displacement obtained is always included in 2 mm margin in all cases (mean 1.03 mm ± 0.34 mm). This parallels the study by Burton et al.,^(1)^ who found mean displacement of 1.2 mm (within 2 mm in 92% cases). In a study by Warrington et al.,^(15)^ the variation of relocation of GTC frame was more in yz plane. This was caused by tilt along the anteroposterior axis which resulted in relatively more error in this direction. Our study also found similar results with systematic and random error being more in this direction. This may be influenced by the condition of upper dentition and the occlusive dental impression. The quality of the dentition of the patient, the experience and skill of the radiation technologist in molding and fixation of the dental impression, and the fitting of the strap are important factors in accuracy of relocation of the frame.

The dosimetric and clinical effects of systematic error are more pronounced than the random error. The margin recipes proposed by Stroom and van Herk prescribe different weight to systematic and random error. Stroom's formula gives three times more weight to systematic error compared to random error. The weight given to systematic error is even more in the van Herk formula. This difference is due to the different dosimetric criteria used for margin calculation. Strrom's formula is calculated to satisfy the criteria that 99% of CTV should get at least 95% dose, whereas van Herk's formula required that for 90% of the patients, the minimum dose should be 95%. For the same systematic and random errors, this leads to a larger margin in the van Herk formula. The van Herk formula is based on a convolution model, which assumes normal Gaussian distribution of systematic and random errors. The underlying assumptions are: homogeneity of the tissue, valid convolution method (number of fractions being sufficiently large), spherical target volume which is large in comparison to set up errors, dose penumbra < 0.5cm, and conformance of the dose distribution to the target. In most clinical settings, the target may not be perfectly spherical and recent studies have shown that the assumption of perfect conformance of the dose distribution to the target may not hold true.^(16)^ Dosimetric margin distribution (DMD) which is the margin between CTV and planned PTV minimum dose isodose surface is more sensitive to setup error than the CTV to PTV margin calculated by the van Herk formula. It is a generalization of the ICRU conformity index. Studies by Gordon et al.^(16)^ showed patients may tolerate larger values of Σ and σ than predicted by the van Herk formula. A smaller CTV to PTV margins based on DMD than those given by the van Herk formula can still achieve the desired level of target coverage depending on the patient anatomy, target site, treatment technique and planning system. In the brain, as the structures are situated intracranially, the organ motion can be considered negligible. Also, since van Herk's formula assumes an isotropic error distribution and we measured the directional errors, van Herk's formula becomes an approximation to the ICRU 62 definition.

## V. CONCLUSIONS

The present study shows that the depth helmet with measuring probe is a clinically viable method for assessing the relocatable accuracy of GTC frame in fractionated stereotactic radiotherapy. The spreadsheet used (obtained from K. E. Burton Department of Oncology, Addenbrooke's Hospital, Cambridge, with kind permission) was found to be very user‐friendly and an easy tool for immediate calculation of translational displacements in mediolateral, craniocaudal and superoinferior directions. The errors of measurements were within ± 2 mm in 99.6%, 99.2%, and 97.6% of cases in mediolateral, anteroposterior and craniocaudal directions, respectively. Systematic, random error and CTV to PTV margin were more along the anteroposterior direction, which may be reduced by quality assurance of the dental impression and skill in mold room techniques. The CTV to PTV margin ranged from 0.93 mm to 1.86 mm by ICRU formula and the mean radial displacement was 1.03 mm. CTV to PTV margin calculated by Stroom's and van Herk's formula exceeded 2 mm in anteroposterior axis only. Fundamental assumptions of van Herk formula based on isotropic error distribution show that conformance of the dose distribution to the target can overestimate the margin and in this case, in view of directional error measurement, van Herk's formula becomes an approximation to the ICRU 62 definition. Therefore, a CTV to PTV margin of 2 mm is a reasonable approximation to compensate for the setup errors.
